# Usability of a website to promote treatment adherence for adults with HIV

**DOI:** 10.17533/udea.iee.v43n3e05

**Published:** 2025-10-18

**Authors:** Aiodelle dos Santos Machado, Marcelo Ribeiro Primeira, Raquel Einloft Kleinubing, Daniel Gonzalo Eslava Albarracin, Tassiane Ferreira Langendorf, Stela Maris de Mello Padoin, Cristiane Cardoso de Paula

**Affiliations:** 1 Nurse, M.Sc. Email: aiodellemachado@gmail.com. https://orcid.org/0000-0002-0128-8793 University Hospital of Bonn Germany aiodellemachado@gmail.com; 2 Nurse, Ph.D. Postdoctoral fellow. Email: mrp_sm@hotmail.com. https://orcid.org/0000-0001-9735-6502 Universidade Federal de Santa Maria Brazil mrp_sm@hotmail.com; 3 Nurse, Ph.D. Temporary Lecturer. Email: raquel_e_k@hotmail.com. https://orcid.org/0000-0002-7448-4699 Universidade Federal de Santa Maria Brazil raquel_e_k@hotmail.com; 4 Nurse, Ph.D. Email: dgeslava@gmail.com. https://orcid.org/0000-0001-7257-4706 Cafam University Foundation Colombia dgeslava@gmail.com; 5 Universidade Federal de Santa Maria Universidade Federal de Santa Maria Brazil tassiane.langendorf@ufsm.br https://orcid.org/0000-0002-5902-7449; 6 Nurse, Ph.D. Permanent Lecturer. Email: stela.padoin@ufsm.br. Corresponding author. https://orcid.org/0000-0003-3272-054X Universidade Federal de Santa Maria Brazil stela.padoin@ufsm.br; 7 Nurse, Ph.D. Permanent Lecturer. Email: cristiane.paula@ufsm.br. https://orcid.org/0000-0003-4122-5161 Universidade Federal de Santa Maria Brazil cristiane.paula@ufsm.br; 8 University Hospital of Bonn, Germany University Hospital of Bonn University Hospital of Bonn Germany; 9 Department of Nursing, Federal University of Santa Maria, Brazil Universidade Federal de Santa Maria Department of Nursing Federal University of Santa Maria Brazil; 10 Cafam University Foundation, Colombia Cafam University Foundation Cafam University Foundation Colombia

**Keywords:** HIV, treatment adherence and compliance, adult, educational technology, biomedical translational science, nursing., VIH, cumplimiento y adherencia al tratamiento, adulto, tecnología educacional, ciencia translacional biomédica, enfermería., HIV, cooperação e adesão ao tratamento, adulto, tecnologia educacional, ciência translacional biomédica, enfermagem.

## Abstract

**Objective.:**

To evaluate a website-type care-educational technology aimed at promoting adherence to antiretroviral treatment in adults living with HIV.

**Methods.:**

This study was based on a Knowledge Translation into Action project developed in two stages: in the first stage, a free-access website was created through collaboration among different research groups; in the second stage, a cross-sectional study was conducted with focus groups and individual interviews with eight participants, using the Assistive Technology Assessment Instrument to evaluate the website’s attributes.

**Results.:**

The website was established as a digital care-educational technology, providing information supported by scientific evidence on self-efficacy, social support, and quality of life. The proposed issues addressed different situations in the lives of people living with HIV, with the objective of fostering treatment adherence. In the overall assessment conducted by the target audience, the website obtained a mean score of 1.88 (minimum 1.1 and maximum 2.0), being classified as adequate. Suggestions received were incorporated through adjustments in design and content structure.

**Conclusion.:**

The website can be used by adults living with HIV and by nursing professionals to promote treatment adherence, contributing to self-care and health education.

## Resumen

**Objetivo.:**

Evaluar una tecnología educativa de cuidado, en formato de sitio web, orientada a promover la adhesión al tratamiento antirretroviral en adultos que viven con VIH.

**Métodos:**

. Estudio basado en un proyecto de traducción del conocimiento en acción que se desarrolló en dos etapas: en la primera se construyó un sitio web, de acceso gratuito, mediante la colaboración de diferentes grupos de investigación; y en la segunda, se llevó a cabo un estudio transversal con entrevistas grupales e individuales a ocho participantes, empleando el Instrumento de Evaluación de Tecnologías Asistenciales para valorar los atributos del sitio web.

**Resultados.:**

El sitio web diseñado constituyó una tecnología digital para la educación sobre el cuidado, que ofreció información respaldada por evidencias científicas sobre autoeficacia, apoyo social y calidad de vida. Las preguntas incluidas son propositivas y relacionadas con distintas situaciones de la vida diaria de las personas con VIH que tienen por propósito favorecer la adhesión al tratamiento. En la evaluación global realizada por el público objetivo, el sitio web obtuvo un promedio de 1.88 (mínimo de 1.1 e máximo de =2.0), clasificado como adecuado. Las sugerencias recibidas se incorporaron mediante ajustes en el diseño y la estructura del contenido.

**Conclusión.:**

El sitio web puede ser utilizado por adultos que viven con VIH y por enfermeros, para promover la adherencia al tratamiento, lo cual puede contribuir al fortalecimiento del autocuidado y al mejoramiento de la atención en el marco de la educación en salud.

## Resumo

**Objetivo.:**

Avaliar uma tecnologia cuidativo-educacional do tipo *website* voltada à promoção da adesão ao tratamento antirretroviral em adultos que vivem com HIV.

**Métodos.:**

Estudo fundamentado em um projeto de tradução do conhecimento em ação, desenvolvido em duas etapas: na primeira, construiu-se um *website* de acesso gratuito por meio da colaboração entre diferentes grupos de pesquisa; na segunda etapa, realizou-se um estudo transversal com entrevistas coletivas e individuais com oito participantes, utilizando-se o Instrumento de Avaliação de Tecnologias Assistenciais para analisar os atributos do *website*.

**Resultados.:**

O *website* desenvolvido constituiu-se em uma tecnologia digital de educação para o cuidado, oferecendo informações sustentadas em evidências científicas sobre autoeficácia, apoio social e qualidade de vida. As questões propostas contemplaram diferentes situações da vida das pessoas que vivem com HIV, com o objetivo de favorecer a adesão ao tratamento. Na avaliação global realizada pelo público-alvo, o *website* obteve média de 1,88 (mínimo de 1,1 e máximo de 2,0), sendo classificado como adequado. As sugestões recebidas foram incorporadas mediante ajustes no design e na estrutura do conteúdo.

**Conclusão.:**

O *website* pode ser utilizado por adultos que vivem com HIV e por profissionais de enfermagem, a fim de promover a adesão ao tratamento, contribuindo para o fortalecimento do autocuidado e para a melhoria da assistência no âmbito da educação em saúde.

## Introduction

Globally, approximately 39 million people are currently living with HIV, of whom 29.8 million are receiving antiretroviral therapy (ART). The Joint United Nations Programme on HIV/AIDS (UNAIDS) has set a global target of ensuring that 35 million individuals are on HIV treatment by 2025.^1^ To this end, the provision of accessible treatment services and equitable access to medicines and other health technologies must be ensured.^1^ In order to guarantee the timely availability of ART, it is essential that individuals are aware of their diagnosis. Accordingly, UNAIDS has proposed that, by 2030, 95% of people living with HIV should know their serological status and, subsequently, be receiving ART with viral load suppression. This strategy is expected to prevent approximately 3.6 million new HIV infections and 1.7 million deaths related to the infection.^1^

In Brazil, between 2012 and 2022, access to ART contributed to a 26.5% reduction in the AIDS-related mortality rate.^2^ However, access to diagnosis and ART alone does not determine treatment adherence, as multiple aspects and factors may be associated with challenges in adhering to ART, including the occurrence of medication side effects, given its long-term use, as well as lifestyle adjustments and behavioral changes.^3^ Access to information through health education mediated by technologies may help mitigate the various factors that hinder adherence.

In this context, adherence to ART is a lifelong and dynamic process, in which challenges can be managed, as the factors influencing adherence vary across different settings.^4^ Considering these circumstances, monitoring and evaluating ART adherence among individuals undergoing treatment is essential.^3^ As a non-pharmacological strategy, the use of tools such as Care-Educational Technologies (CET) is recommended. These tools aim to strengthen individuals’ knowledge and foster meaningful learning through human empowerment regarding their health condition and the environment in which they live.^5^^-^^6^ Within the scope of health education, the application of such educational resources by nursing professionals may contribute to improved ART adherence.^7^

Thus, the role of nurses is strategic for planning and providing care, as well as for promoting self-care and shared responsibility in individual health, with the introduction of CET acting as mediators of health literacy. This adds to the importance of nursing in the development, implementation, and execution of public policies in the context of HIV infection.^8^ The Brazilian Board of Nursing, in partnership with the Ministry of Health, has played a fundamental role in establishing regulations that guide and strengthen nursing practices in combating sexually transmitted infections. The aim is to provide nursing professionals with the necessary tools to manage the care of individuals affected by infections under the responsibility of the Department of HIV/AIDS, Tuberculosis, Viral Hepatitis, and Sexually Transmitted Infections.^9^

Considering that the tools and technologies implemented in the context of HIV infection must address the local reality, as well as the target audience to be accessed,^10^ this study is justified by the need to evaluate the target audience of a CET. Furthermore, it is worth noting the convergence with the 3^rd^ Sustainable Development Goal (SDG) proposed by the United Nations (UN) in the 2030 Agenda.^11^ Therefore, the objective of this study was to evaluate a website-based care-educational technology aimed at promoting adherence to antiretroviral treatment among adults living with HIV.

## Methods

This is a Knowledge-to-Action (KTA) project^12^^,^^13^ structured around two cycles: the knowledge creation cycle and the knowledge-to-action cycle. In this study, during the knowledge creation cycle, an informational website prototype was developed with online access for use on smartphones, tablets, laptops, and desktop computers. In the knowledge-to-action cycle, the principle of adapting knowledge to the local context was followed, with an investigation conducted to evaluate the website among adults living with HIV infection.

For the website prototyping, the textual content underwent expert validation, while the visual content was conceptualized by the research team and produced by a professional illustrator in a previous study.^14^ The purpose of the tool is to foster adherence to antiretroviral therapy among adults living with HIV, thereby translating scientific evidence into a practical knowledge tool. Accordingly, the website is classified as a CET, as it constitutes a non-pharmacological strategy designed to integrate care and education. The website structure was organized into three thematic sections (Table 1).

**Table 1:** Website content structure

Structure	Page	Title and web layout
Home	1	Presentation of the *Conviva* brand; purpose of the website and buttons to access the content or learn about the creative team.
Section 1	2	Context of the content covered; buttons for concepts of adherence, social support, self-efficacy, and quality of life; button to continue to ‘life situations’; button to return to home page and button to continue to first concept.
	3	Adherence concept; buttons to return to home page, return to previous menu, and continue to next concept.
	4	Social support concept; buttons to return to home page, return to previous menu, and continue to next concept.
	5	Self-efficacy concept; buttons to return to home page, return to previous menu, and continue to next concept.
	6	Quality of life concept; buttons to return to home page, return to previous menu, and continue to ‘life situations’ menu.
Section 2	7	Context of the content; menu with links that redirect to Life Situations (LS) pages from LS1 to LS11; buttons to return to home page, return to concepts, and continue to first LS.
	8	LS1 Stigma and Prejudice; buttons to return to home page, return to LS menu, and continue to next LS.
	9	LS2 Antiretroviral Medicines; buttons to return to home page, return to LS menu, and continue to next LS.
	10	LS3 Side Effects; buttons to return to home page, return to LS menu, and continue to next LS.
	11	LS4 Negative Emotions; buttons to return to home page, return to LS menu, and continue to next LS.
	12	LS5 Stigma and Prejudice; buttons to return to home page, return to LS menu, and continue to next LS.
	13	LS6 Misinformation or Denial of Health Condition; buttons to return to home page, return to LS menu, and continue to next LS.
	14	LS7 Communication Challenges between Users and Healthcare Team; buttons to return to home page, return to LS menu, and continue to next LS.
Section 3	15	LS8 Healthy Habits that Improve Health and Quality of Life; buttons to return to home page, return to LS menu, and continue to next LS.
	16	LS9 Concerns about Effects of Antiretroviral Medicines; buttons to return to home page, return to LS menu, and continue to next LS.
	17	LS10 Concerns about Health, Treatment, and Fear of Death; buttons to return to home page, return to LS menu, and continue to next LS.
	18	LS11 Adherence to Prescription Makes the Body Stronger and Healthier; buttons to return to home page, return to LS menu, and continue to credits.
Credits	19	Authors; Illustrator; Programmers; Text review team; Support team; Funding; Button to return to home page.

**Figure 1:**
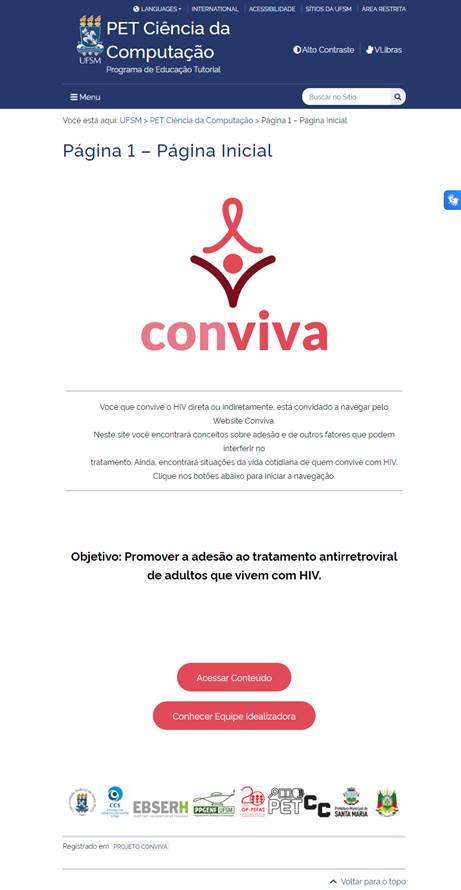
Home page of the *Conviva* website

During the development stage of the website’s interface and visual identity, both textual and visual contents were integrated in partnership with the Tutorial Education Program in Computer Science team, between March and June 2023. Considering that the website would be made available under an institutional domain, linked to its official website, the platform selected for interface development was WordPress®, a free and open-source web content management system. The visual identity was designed by the same illustrator responsible for producing the images. Aiming to convey the message about learning to live with antiretroviral treatment, the design depicts a person with open arms (happy and content with life), above which appears the universal symbol of the fight against the HIV epidemic. In the logo (Figure 1), the word *VIVA* (live, in Portuguese) is highlighted to reinforce the idea of getting used to and adapting to the new health care routine.

The prototype was reviewed by a panel of experts consisting of members of the research group and representatives from healthcare services and municipal management, including six nurses and one physician. This step follows the recommendations from the KTA framework authors, who emphasize that knowledge adaptation should consider the participation of diverse audiences to achieve greater success in its use by the end user.^13^ To start the knowledge-to-action cycle, more specifically at the stage of adapting knowledge to the local context, an evaluation was conducted with adults living with HIV. At this stage, the aim was to identify contextual particularities and efficiency in terms of acceptance in an environment with particular characteristics.^13^ The study was conducted in two specialized healthcare services and a Support Center for People Living with HIV, located in a medium-sized city in the central region of the state of Rio Grande do Sul, Brazil. The sample for the website evaluation consisted of eight participants who met the inclusion criteria: being 18 years of age or older, living with HIV infection, receiving follow-up care in the participating services, being literate, and not presenting visual impairment or other conditions that would prevent the visualization and reading of the material provided. 

Two data collection strategies were employed: focus group and individual interviews. The use of both strategies was necessary due to the limited participation of people living with HIV in focus groups, as the topic is sensitive and individuals often prefer to maintain confidentiality of their diagnosis because of the stigma associated with the epidemic. Sections 1 and 2 were assessed in the focus groups, while in the individual interviews all sections were evaluated. For both strategies, the Assistive Technology Assessment Instrument^14^ and sociodemographic characterization questions were applied.

The focus groups were conducted in two meetings: 1) evaluation of Section 1 (pages 1-6), which covers the concepts addressed throughout the website, such as adherence, social support, self-efficacy, and quality of life; and 2) evaluation of Section 2 (pages 7-14), which refers to Life Situations (LSs 1-7) of stigma and prejudice, antiretroviral medicines, side effects, negative feelings, misinformation or denial of health condition, and communication challenges. In the five individual interviews, participants completed the full assessment (Sections 1, 2, and 3), and after the website was presented, they were free to make suggestions. After the presentation of the full content, participants completed the Assistive Technology Assessment Instrument. Subsequently, they completed the assessment of Section 3 (pages 15-18), which refers to Life Situations (LSs 8-11) of healthy habits that improve health and quality of life, concerns about effects of antiretroviral medicines, concerns about health, and adherence to prescription makes the body stronger and healthier. 

For the analysis of the instrument data, the results were entered into a Microsoft Excel® spreadsheet and exported to the Statistical Package for the Social Sciences (SPSS®) software. Mean scores were calculated per item and per attribute, in addition to the overall mean based on the target audience’s assessment. Items or attributes with a mean of zero (0) were classified as inadequate; those with means between 0.1 and 1.0 were classified as partially adequate; and those with means between 1.1 and 2.0 were classified as adequate.^16^ The information collected from audio transcripts was compiled, and suggestions were incorporated into adjustments to the website. Data collection was conducted after the project received approval by the Research Ethics Committee, under Registration No. 67915823.0.0000.5346.

## Results

The website designed to promote adherence to antiretroviral treatment for adults living with HIV infection (Figure 1) is accessible at https://www.ufsm.br/pet/ciencia-da-computacao/conviva-1. Finally, the website was registered as Technical Work under No. 712146145.

Eight adults living with HIV infection, aged between 28 and 59 years, participated in the study, five of whom were male and three female. Regarding self-reported race, four participants identified as white, one as brown, and three as black. In terms of educational level, two had completed elementary school, one had completed high school, two had completed higher education, and three had not completed higher education. The time since HIV diagnosis ranged from 2 to 23 years, while the duration of treatment ranged from 6 months to 19 years. Concerning the overall assessment according to the instrument applied, the website achieved a mean score of 1.88 and was considered adequate by the participants, according to the classification scale ranging between 1.1 and 2.0^16^ (Table 2).

**Table 2:** Website assessment criteria by attribute, Brazil, 2024

Attribute	Item	n*	Min†	Max‡	Mea§	Mea§ by Attribute
Attribute 1 Interactivity	The content is suitable for your needs	8	1	2	1.75	1.88
Offers interaction and active involvement in the educational process	8	1	2	1.75
Enables access to the topics covered	8	2	2	2.00
Allows users to operate it autonomously	8	2	2	2.00
Attribute 2 Objectives	Promotes learning of the content covered	8	2	2	2.00	1.81
	Encourages the learning of new concepts and facts	8	1	2	1.75	
	Enables users to easily find information	8	1	2	1.63	
	Employs an appealing presentation strategy	8	1	2	1.88	
Attribute 3 Relevance and Effectiveness	Provides adequate resources for use	8	2	2	2.00	1.91
	Fosters interest in its use	8	1	2	1.88	
	Encourages behavioral change	8	1	2	1.88	
	Presents the content covered across various contexts	8	1	2	1.88	
Attribute 4 Clarity	Presents information in a clear and simple manner	8	1	2	1.88	1.94
	Allows reflection on the content presented	8	2	2	2.00	
	Overall mean					1.88

Note: n* = number; † = Minimum; ‡ = Maximum; § = Mean.

With regard to the assessment of the website’s attributes, *clarity* received the highest average rating, whereas *objectives* obtained the lowest. Although the overall assessment indicated that the website was suitable for use, the suggestions for improvements made by participants during the interviews were either incorporated into the website or, when not feasible, justified by system or operational limitations (Table 3).

**Table 3:** Suggestions and adjustments following end-user assessment of the website

Suggestions	Adjustments
Color changes on page 7.	Changes included.
Remove explicit reference to diagnosis on home page design.	
Inclusion of reporting channels for prejudice-related issues.	
Page 12: highlight in bold the link to the Rights page.	
Page 10: The ‘continue’ button should be adjusted from ‘next concept’ to ‘next situation’.	
Expand coverage of legal issues (rights of people living with HIV).	Link to external page with official information already available.
Include icons/emojis next to LS titles.	Changes not included due to system/operational limitations.
Turn the website into an interactive app where users can enter their health information.	
Insert content into an app instead of a website.	
Provide additional information on who may access PrEP and PEP	Changes not implemented due to requirement for modification of previously validated content.
Inclusion of information on modes of transmission.	
Inclusion of information on risk behaviors for infection.	
Emphasize the concept of ‘undetectable = untransmittable,’ particularly for HIV-negative individuals.	
Include information on where to seek help (mental health).	Change not implemented due to requirement for service-specific information and variability in local mental health care.

## Discussion

In the study in question, the website was considered adequate by adults living with HIV infection with respect to its interactivity, objective, relevance and effectiveness, and clarity.^15^ These findings indicate the website’s positive reception by the target audience and suggest that autonomy in free access may contribute favorably to the promotion of self-care. The evaluation conducted by users is essential, as they possess unique knowledge, perspectives, and experiences that can influence the quality, appropriateness, and usability of the product.^17^ Ensuring consistency among the theme, structure, type of product, and target audience is fundamental to achieving the intended educational objectives.^18^ Furthermore, in addition to the overall mean obtained from the *Conviva* website assessment tool, the attributes of the educational resource should also be examined individually, as they highlight both opportunities for improvement and weaknesses identified during the evaluation process.

The *interactivity* attribute assesses whether the subject is actively and participatively engaged in the educational process and refers to the possibility of the user finding information according to their interests and the pace of their occupational activities. Interaction stimulates decision-making and learning processes, thereby reinforcing knowledge acquisition.^15^ Thus, the evaluation of the website’s interactivity as adequate suggests that its use can enhance end-user health literacy and, consequently, promote self-care. A meta-review including 55 randomized clinical trials with different e-health interventions demonstrated that these approaches can be effective in promoting adherence to ART and improving health outcomes in people living with HIV. Within the review, *m-Health* was the most studied component, comprising the use of mobile devices, such as smartphones and tablets, to promote health and self-care, including health education platforms.^19^

Furthermore, a systematic review found that participants who used the software alongside their regular consultations over an 18-month period were 12% more likely to achieve perfect adherence compared to those receiving standard care. However, no significant differences were observed in the short term.^20^ In this sense, the decision to develop the *Conviva* website is consistent, as it represents a continuously accessible technological resource.

The *objectives* attribute refers to the purposes and goals that should be achieved through the use of the proposed technology. This attribute obtained the lowest mean score among those evaluated (1.81), although it was still considered adequate for its intended purpose. In addition, the *clarity* attribute relates to whether the information presented is easy to understand, that is, whether the content is conveyed in a clear and accessible manner.

Considering that the purpose of the *Conviva* website is to promote adherence to antiretroviral therapy among adults living with HIV, the positive evaluation of the *objectives* and *clarity* attributes, particularly regarding the accessible presentation of information, supports its potential use in health education. The adoption of clear information conveyed through appropriate language has been a central objective in the development of educational resources, as it contributes to improving health literacy among the target audience of such technologies.^21^

Furthermore, the *clarity* attribute refers to the capacity of the educational intervention to foster reflection on adherence-related content. In this study, participants raised questions concerning their health condition and lived experiences, which demonstrated convergence with the LSs presented on the website. Reflections on self-efficacy for adherence also emerged, as illustrated by one participant who described the daily effort required to maintain medication use and linked this difficulty to a perception of being unable to manage treatment independently, compounded by issues such as prejudice toward their own serological status. Given the importance of adherence to antiretroviral therapy, it is essential to examine the factors that may compromise this process. Expectations of self-efficacy can directly influence adherence, as perceptions of one’s own competence affect treatment execution and performance, potentially leading to interruptions or discontinuation of therapy.^22^

The *relevance and effectiveness* attribute refers to the significance of the educational resource and its capacity to generate impact, motivation, and interest,^15^ having obtained a mean score of 1.91 in the present study. *Relevance* is particularly noteworthy, as it encompasses the influence of technology on the target audience, both in promoting adherence to ART and in fostering expectations of self-efficacy for treatment, thereby contributing to quality of life and strengthening social support. Moreover, free access to information is considered an important factor in bridging knowledge gaps and, consequently, in reducing vulnerabilities. 

The LSs presented on the website, based on perceived social support, stimulate reflection on the presence or absence of social support, which tends to directly influence adherence to ART. The social and emotional consequences of living with HIV pose challenges that affect relationships in the workplace, family, and community.^23^ Support networks perceived as satisfactory represent essential resources in the care process. Conversely, family abandonment can undermine self-esteem and lead to social isolation, thereby compromising adherence,^23^ as stigma and prejudice within family relationships further diminish perceived social support.^24^

The stigma associated with HIV infection can manifest in different forms, including internalized, anticipated, and enacted stigma. One strategy to mitigate stigma is adherence to antiretroviral therapy, which reduces the fear of death by providing greater life expectancy and improved health, while also contributing to the restoration of one’s social value. Access to health services and higher-quality care can further reduce isolation and strengthen social support. Successful treatments, reflected in healthier bodies, challenge the normalization of stigma toward people living with HIV.^25^ Although digital and infotainment interventions have shown mixed results in reducing stigma, it remains essential to promote education among society, health professionals, and patients themselves to help reduce prejudice.^26^ Moreover, factors related to economic, social, and cultural vulnerabilities, alongside the availability of psychological support, play a crucial role in reducing stigma and enhancing the quality of life of people living with HIV.^27^ These aspects are addressed within the website content and applied in the LSs, comprising the structure of the technology.

The use of educational technologies to promote adherence to antiretroviral therapy has been supported by evidence demonstrating their effectiveness. A systematic review assessing interventions designed to improve self-management of antiretroviral therapy among adults living with HIV concluded that technology-assisted strategies are effective in promoting adherence.^28^ In this study, one factor that may hinder access to information on the website, as reported by participants, was the presence of technical terms, alongside the need to adapt the resource for individuals who are illiterate or have visual impairments. These findings reinforce the premise that educational technologies must be designed in accordance with the characteristics of the target audience to achieve their intended purpose.^29^

Since the quality of life of people living with HIV is positively associated with adherence to antiretroviral therapy,^30^ and this relationship is understood to be bidirectional, it is important that tools designed to promote adherence also address aspects related to quality of life.^31^ Strategies to foster treatment adherence, whether through educational technologies or other available resources, should therefore incorporate key elements of this cycle, including quality of life, self-efficacy, social support, stigma, and prejudice.

The limitations of this study relate to the difficulty of accessing the target audience, underscoring the need to promote awareness to minimize prejudice, stigma, and discrimination against people living with HIV. The *Conviva* website was evaluated as suitable for this population and, as a free digital tool, was reported to facilitate easy access to information. Moreover, the incorporation of evidence-based content allows health professionals to employ it as a resource for health education and to support adherence to HIV treatment. The website met expectations regarding its design and the attributes of interactivity, objectivity, relevance and effectiveness, and clarity. Within the KTA framework, this educational technology demonstrates the translation of complex knowledge into accessible formats, enabling engagement of the target audience and expanding access to evidence-based information. In doing so, it helps bridge the gap between the human right to information and the pursuit of health equity. Finally, we recommend its use in local contexts, with the possibility of adapting it to other national and international settings, given that the challenge of adherence is a global issue.

Funding. This work is derived from a master’s thesis entitled “Website for Promoting Adherence to Antiretroviral Therapy among Adults Living with HIV: Evaluation by the Target Audience.” The project was funded through a joint call for proposals issued by the Brazilian National Council for Scientific and Technological Development (CNPq) and the Brazilian Hospital Services Company (EBSERH). 
